# Impact of dipeptidyl peptidase-4 inhibitors on serum adiponectin: a meta-analysis

**DOI:** 10.1186/s12944-016-0372-7

**Published:** 2016-11-23

**Authors:** Xin Liu, Peng Men, Yuhui Wang, Suodi Zhai, George Liu

**Affiliations:** 1 Institute of Cardiovascular Sciences, School of Basic Medical Science, Peking University Health Science Center, 38 Xueyuan Road, Beijing, 100191 China; 2 Department of Pharmacy, Peking University Third Hospital, Beijing, China

**Keywords:** Adiponectin, Dipeptidyl peptidase-4 inhibitors, Sitagliptin, Vildagliptin, Type 2 diabetes mellitus

## Abstract

**Background:**

Adiponectin, an adipose-specific protein, is negatively correlated with pro-atherogenic low-density lipoprotein cholesterol (LDL-C) and other cardiovascular risk factors such as insulin resistance. Therefore, low levels of adiponectin are associated with a higher risk for diabetes and cardiovascular disease. Dipeptidyl peptidase-4 inhibitors (DPP4i) have been used for the treatment of type 2 diabetes mellitus (T2DM) as reversible inhibitors through interacting with DPP4 substrate and increase serum incretins such as glucagon-like peptide-1 (GLP-1). The present study aimed to evaluate the effect of DPP4i on serum adiponectin in T2DM patients.

**Methods:**

The PubMed, Embase, and Cochrane library databases were searched from inception to February 2016. Randomized controlled trials, evaluating the DPP4i (sitagliptin and vildagliptin) versus comparator (placebo or active-comparison), in T2DM patients with duration of ≥ 12 weeks, were identified. Weighted differences in means of adiponectin levels were calculated by using a fixed or random-effects model.

**Results:**

Ten randomized controlled trials, including 1,495 subjects, were identified. Compared with placebo, DPP4i (sitagliptin and vildagliptin) treatment significantly elevated adiponectin levels by 0.74 μg/mL (95% confidence interval [CI], 0.45 to 1.03) relative to that using an active-comparison by 0.00 μg/mL (95% CI, −0.57 to 0.56). Compared with active-comparison, vildagliptin treatment increased adiponectin levels by 0.32 μg/mL (95% CI, −0.01 to 0.65), whereas sitagliptin treatment decreased adiponectin levels by −0.24 μg/mL (95% CI, −1.07 to 0.58). Trials examining effects of other DPP4i were not found.

**Conclusions:**

Sitagliptin and vildagliptin increased serum adiponectin levels and had no stronger effect than traditional oral antidiabetic drugs. Further trials with larger sample size are needed to confirm the results and investigate the association between serum adiponectin levels and treatment of other DPP-4 inhibitors.

**Trial registration:**

Registration No in PROSPERO: CRD42016037399.

## Background

Obesity is strongly associated with type 2 diabetes mellitus (T2DM) and cardiovascular mortality [1]. Therefore, efforts have been made to better understand mechanisms underlying the etiology of obesity and cardiovascular disease. Adipocytes are not only energy-storing cells, but also secrete bioactive substances such as adiponectin [2, 3]. Adiponectin is an adipose-specific collagen-like protein that adheres to injured arterial endothelial walls [1]. The adiponectin gene is located on chromosome 3q27 and has been linked to metabolic syndrome and T2DM [4]. Adiponectin is often reduced along with insulin resistance progression in T2DM [5].

Previous studies have indicated that adiponectin possessed favorable anti-diabetic and anti-atherogenic activities [6, 7]. Adiponectin levels are lower in patients with T2DM, which is a common co-morbidity of obesity [8]. Adiponectin is positively correlated with insulin sensitivity in humans and animals [9], and negatively correlated with myocardial infarctions [10]. Plasma adiponectin levels are markedly lower in patients with coronary artery disease compared with age- and BMI-matched controls [11]. Adiponectin also has a cardioprotective effect during ischemia-reperfusion injury [12]. Hypoadiponectinemia has been related to cardiac hypertrophy [13].

Increased adiponectin levels improve atherosclerosis in patients with T2DM or insulin resistance. Thiazolidinediones, which are useful glucose-lowering agents that have off-target or unwanted side effects, increase adiponectin levels in humans and mice [14, 15]. GLP-1, mainly in the form of incretin, increases adiponectin secretion in obese mice [16]. Dipeptidyl peptidase-4 inhibitors (DPP4i), or gliptins, lower glucose levels during fasting and post-prandial states via preventing the inactivation of incretin hormones [17, 18]. Clinical studies have indicated that gliptins improve pancreatic β-cell function in T2DM patients [19, 20]. In animals, administration of DPP4i increases diet-induced decline in adiponectin levels [21]. Investigations on the effect of gliptin on plasma adiponectin in T2DM patients are limited, and the available data remains controversial [22–24]. In 2008, the U.S. Food and Drug Administration and the European Medicines Agency simultaneously required a demonstration of cardiovascular safety for all new glucose-lowering medications. Based on the reported benefits of higher adiponectin levels, adiponectin may be potentially used as a novel surrogate cardiovascular indicator and biomarker for antidiabetic agents. Therefore, to better understand gliptin activity on adiponectin, a meta-analysis of clinical trial data on DPP4i was conducted in adult T2DM patients.

## Methods

### Search strategy

In consultation with a medical librarian, we established a search strategy for the PubMed, Embase, and Cochrane Library using the following query terms: “sitagliptin,” “vildagliptin,” “teneligliptin,” “saxagliptin,” “linagliptin,” “anagliptin,” or “alogliptin”. Randomized controlled trials published in English (from inception to February 2016) were included in the analysis. Two researchers independently searched for studies and any disagreement was resolved through discussion.

### Study selection

Studies were included in the meta-analysis when they described a randomized clinical trial involving T2DM patients that lasted at least 12 weeks and compared a drug to a placebo or an active comparator other than a DPP4i. Exclusion criteria consisted of nonhuman studies, review articles, observational studies or case reports, non-glucose-lowering therapies, duplicate publications, short-term studies (therapies duration of < 12 weeks) and studies in which changes in adiponectin levels was not reported or could not be calculated. The corresponding authors were contacted when clarification on specific aspects of the study was required. The inclusion and exclusion criteria were objectively evaluated by two reviewers.

### Data extraction and outcomes

Two researchers (X.L. and P.M.) extracted data and used a standardized form of recording study information (author, publication year, sample size, trial duration and controls) and population characteristics (body mass index, age, sex, body weight, and baseline levels of glycosylated hemoglobin and adiponectin). Disagreement was resolved by discussion. The primary outcome was assessed using the difference between the final and baseline adiponectin levels. All included studies recorded mean adiponectin levels compared to that using placebo or active comparators.

### Quality appraisal

Two researchers independently evaluated the quality of each clinical trial according to the Cochrane risk of bias tool [25]. This assessment included low, unclear and high risk for bias based on random sequence generation, allocation concealment, blinding of participants, blinding of outcome assessment, incomplete outcome data, selective reporting and other bias. Discrepancies were resolved by discussion or consultation of a third reviewer.

### Statistical analysis

Statistical analysis were performed using Review Manager (Revman 5.1; Cochrane Collaboration) and STATA 12.0. Pooled weighted mean differences (WMD) and 95% confidence intervals (CIs) were calculated using the Mantel-Haenszel (M-H) fixed effects model when there was no evidence of significant heterogeneity for outcomes or a random effects model when significant heterogeneity for outcomes was present. Heterogeneity among studies was assessed using a Chi-squared test and quantified using Higgins I^2^. To account for low statistical power of the Chi-squared test for heterogeneity, *p* = 0.10 was considered insignificant. I^2^ ranging from 0 to 50% indicated that there was no important heterogeneity, and I^2^ values ranging from 50 to 100% suggested major heterogeneity, which was further explored using sensitivity analysis. Publication bias was examined by Egger’s tests if there were at least five studies for each outcome.

## Results

### Trial characteristics and quality

**Fig. 1:**
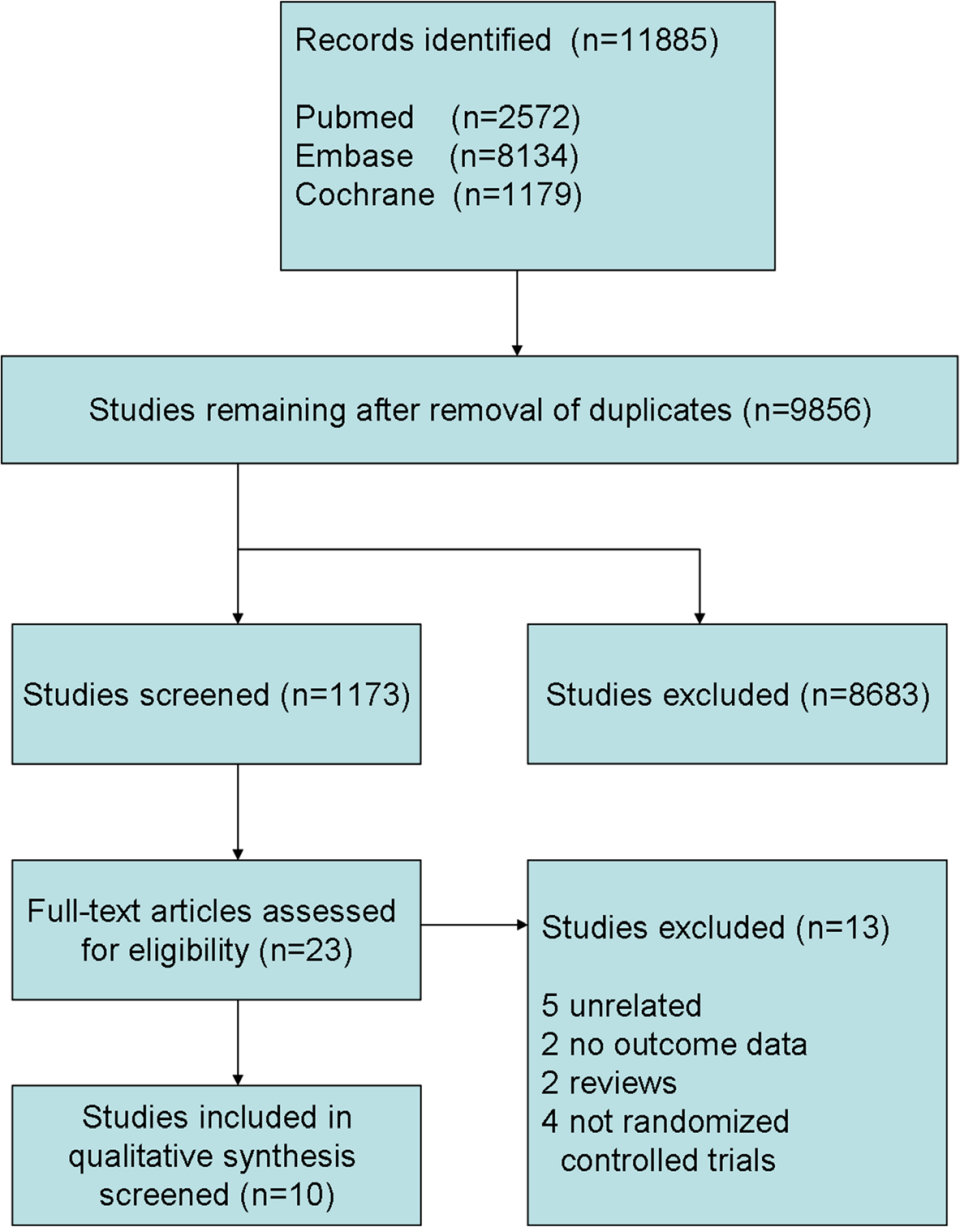
Flow chart of studies included in the meta-analysis

**Fig. 2:**
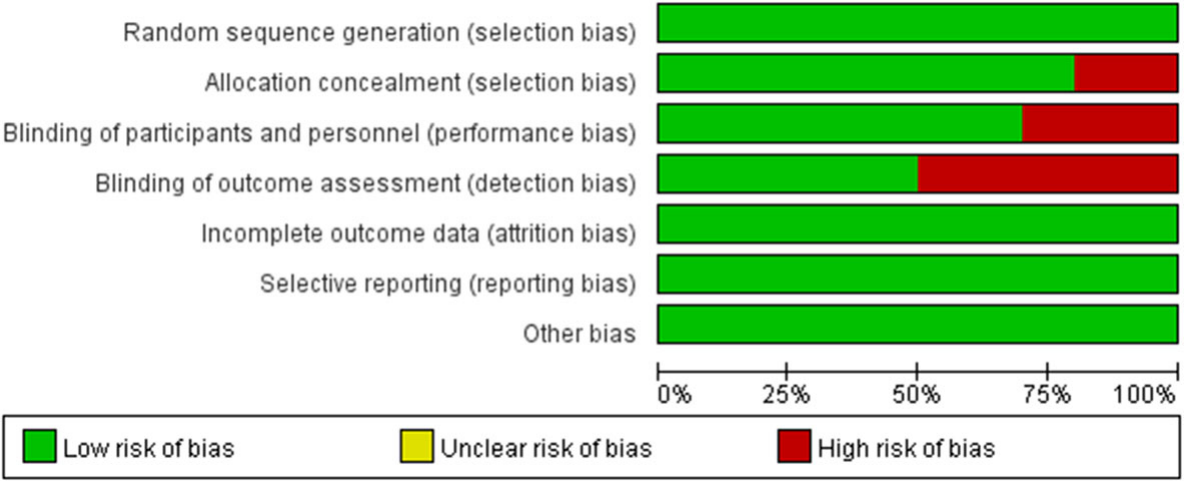
Study quality as evaluated by Cochrane scores

Initially, 11,885 citations were found to meet the inclusion criteria. A study inclusion flowchart was presented in Fig. 1. After removal of inappropriate studies, ten randomized controlled trials including 1,495 subjects were identified, four studies were performed in Italy, one in Germany, and five in Japan. Characteristics of the 10 intervention studies are shown in Table 1. Trial quality was evaluated by using the Cochrane risk of bias tool (Fig. 2). Five studies fully fit the seven factors. Two studies were adequate for six of the seven factors, one study was adequate for five of the seven factors and two studies were adequate for four of the seven factors.

**Table 1:** Characteristics of the 10 studies included in the meta-analysis

Studies	Patients (N)	Treatment	Diabetes duration (months)	Adiponectin (μg/mL)	HbA_1c_ (%)	Treatment duration (months)	Patient characteristics			
							BMI (kg/m^2^)	Age (years)	Male (N)	Weight (kg)
Derosa, 2012 [22]	91	sitagliptin + metformin	5.8	5.0	8.1	12	28.1	55.9	42	78.4
	87	placebo + metformin	5.4	5.2	8.0		28.9	54.8	44	78.6
Derosa, 2010 [23]	75	sitaliptin + pioglitazone	60	5.4	8.5	12	27.9	57	37	78.7
	76	metformin + pioglitazone	72	5.3	8.4		27.7	58	39	77.3
Forst, 2013 [45]	22	vildagliptin + metformin	100.8	5.0	7.41	6	Not available	Not available	Not available	99.3
	22	glimepiride + metformin	73.2	5.6	7.28		Not available	Not available	Not available	93.7
Mikada, 2014 [47]	14	sitagliptin	91.2	6.5	7.45	6	28.8	59.2	11	76.8
	14	miglitol	111.6	7.4	6.90		29.5	58.7	11	81.4
Hibuse, 2014 [48]	16	sitagliptin	57.6	6.7	7.5	3	24.9	63	9	Not available
	10	sulfonylurea and/or biguanide	45.6	4.6	7.8		28.1	56	6	Not available
Takeshita, 2015 [49]	30	sitagliptin	86.4	4.5	6.7	4	24.5	61.0	18	62.5
	30	mitiglinide	145.2	5.0	6.9		24.2	65.8	19	63.2
Derosa, 2012 [50]	84	vildagliptin + metformin	6.1	5.2	8.1	12	27.9	54.2	42	76.9
	83	placebo + metformin	6.3	5.4	8.2		27.8	52.4	43	78.5
Derosa, 2014 [51]	86	vildagliptin	6.9	4.8	7.9	6	27.9	59.8	42	77.8
	81	glimepiride	6.7	4.5	7.8		27.7	56.8	36	77.2
Takeshita, 2015 [52]	58	vildagliptin	Not available	3.6	8.1	3	24.5	Not available	36	63.2
	54	liraglutide	Not available	3.8	8.0		25.4	Not available	35	65.8
Shimoda, 2014 [53]	25	sitagliptin	Not available	7.0	7.3	3	24.9	63.9	16	Not available
	25	glimepiride	Not available	7.6	7.5		25.3	62.4	15	Not available

### Efficacy outcomes

**Fig. 3:**
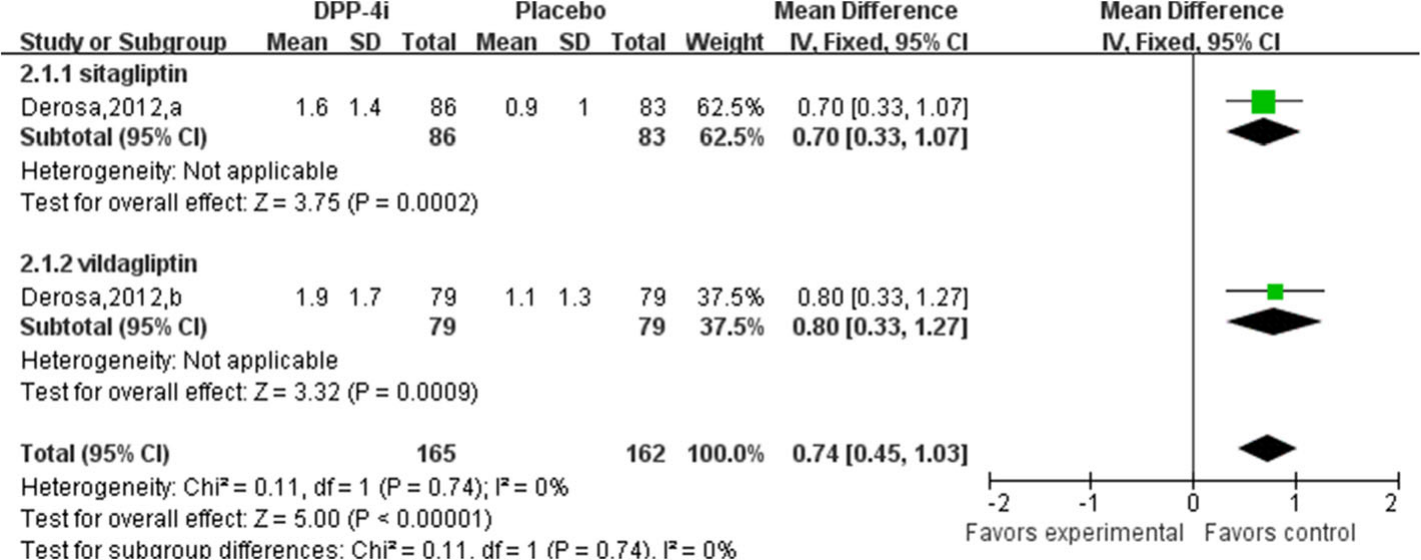
Meta-analysis of the effect of DPP4i treatment versus placebo on serum adiponectin levels

**Fig. 4:**
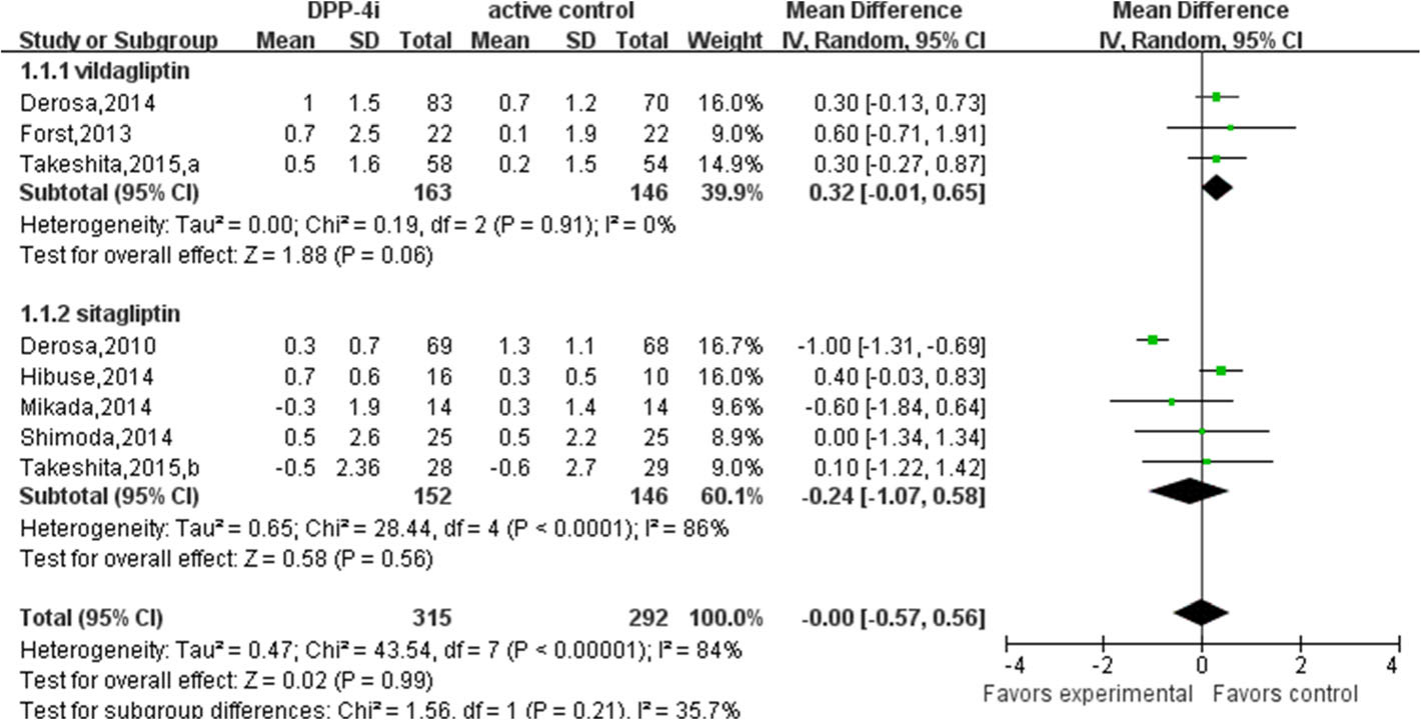
Meta-analysis of the effect of DPP4i treatment versus active comparator on serum adiponectin levels

Results of the analysis of gliptin-adiponectin efficacy are presented in Figs. 3 and 4. Compared with placebo group, DPP4i (sitagliptin and vildagliptin) treatment significantly elevated adiponectin levels by 0.74 μg/mL (95% confidence interval [CI], 0.45 to 1.03; *p* = 0.74; I^2^ = 0%; weighted mean baseline adiponectin: 5.10 ± 0.90 μg/mL *vs.* 5.30 ± 1.15 μg/mL) relative to that using an active comparator by 0.00 μg/mL (95% CI, −0.57 to 0.56; *p* < 0.01; I^2^ = 84%; weighted mean baseline adiponectin: 5.05 ± 2.12 μg/mL *vs.* 5.10 ± 2.01 μg/mL). Compared to active comparator, vildagliptin increased adiponectin levels by 0.32 μg/mL (95% CI, −0.01 to 0.65; *p* = 0.91; I^2^ = 0%; weighted mean baseline adiponectin: 4.40 ± 2.18 μg/mL *vs.* 4.41 ± 1.98 μg/mL), whereas sitagliptin decreased levels by −0.24 μg/mL (95% CI, −1.07 to 0.58; *p* < 0.01; I^2^ = 86%; weighted mean baseline adiponectin: 5.71 ± 2.06 μg/mL *vs.* 5.76 ± 2.03 μg/mL). Trials that examined the effect of other DPP4 inhibitors were not found.

### Heterogeneity and publication bias

**Fig. 5:**
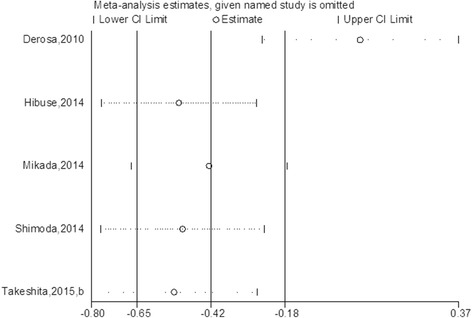
Sensitivity analysis of the studies reporting the effect of sitagliptin comparing with active control on serum adiponectin levels

**Fig. 6:**
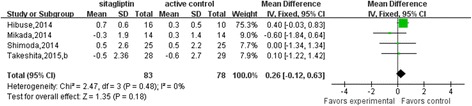
Meta-analysis of the effect of sitagliptin treatment versus active comparator on serum adiponectin levels

A significant heterogeneity was observed in the sitagliptin-active comparator group and a sensitivity analysis was then performed in Fig. 5, which indicated that Derosa’s study had a significant effect on the result. Heterogeneity was not significant after particular study was excluded from the analysis (*p* = 0.48; I^2^ = 0%), and in turn showed that sitagliptin had no stronger effect on serum adiponectin levels compared to traditional oral antidiabetic drugs (0.26 μg/mL, 95% CI, −0.12 to 0.63; Fig. 6). According to Egger’s test, no publication bias was observed among the five sitagliptin studies (*p* = 0.082).

## Discussion

In current meta-analysis, the effect of DPP4i on adiponectin levels in T2DM patients was evaluated based on 10 clinical trials. A pooled efficacy estimate from those trials indicated that administration of sitagliptin and vildagliptin compared to placebo resulted in an increase in serum adiponectin levels, yet was not superior to that of active comparators.

T2DM is positively correlated with morbidity and mortality of diabetic vasculopathies and cardiovascular risks, including microangiopathies (e.g., renal failure and blindness) and macroangiopathies (atherosclerosis). Cardiovascular disease is a major cause of mortality in T2DM patients, and insulin or sulphonylureas effectively reduces the risk for microvascular complications, but not macrovascular events [26]. Of note, some studies have indicated that single and intensive glucose-lowering therapies might be less favorable to cardiovascular endpoints in T2DM [27, 28]. Thus, antidiabetic therapy must be performed on the basis of future cardiovascular aspects.

Body fat distribution, especially visceral fat accumulation, is an early sign of obesity-related disorders such as cardiovascular disease and is associated with atherosclerosis via dysfunctional adipocytes and downregulated production of protective adipocytokines such as adiponectin. Obesity also decreases adiponectin sensitivity by downregulating the expression of AdipoR1 and AdipoR2 adiponectin receptors, which in turn leads to insulin resistance [29].

Adiponectin has an insulin-sensitizing effect by activating adenosine monophosphate activated protein kinase (AMPK) signaling through binding to AdipoR1 and AdipoR2. Then, AMPK stimulates cellular metabolism and enhances glucose uptake, fatty acid oxidation, and glucose utilization, thereby causing an increase in insulin sensitivity [30]. Adiponectin prevents atherosclerosis by inhibiting the expression of monocyte adhesion molecules and endothelial synthesis of pro-inflammatory chemokine by inactivating nuclear factor-kappaB [31, 32] as well as suppressing proliferation of vascular smooth muscle cells by targeting extracellular signal-regulated kinase [33].

Adiponectin regulates inflammatory responses in atherosclerotic lesions by interacting with IL-10 and upregulating the expression of tissue inhibitor of metalloproteinase-1 [34]. In apoE-deficient mice, adenovirally-mediated increases in adiponectin levels inhibit the progression of atherosclerotic lesions by downregulating VCAM-1 and SR-A [35, 36]. Adiponectin diminishes infarct size, apoptosis and inflammatory cytokine in ischemia-reperfusion models through both AMPK- and COX-2-dependent mechanisms [12]. Adiponectin improves cardiac hypertrophy by inhibiting hypertrophic signaling in the myocardium through the activation of AMPK and extracellular signal-regulated kinase (ERK).

PPAR-γ agonists exhibit favorable effects on cardiovascular outcomes in T2DM patients [37, 38]. Thiazolidinediones, which are synthetic PPAR-γ ligands, stimulate serum adiponectin by inducing the expression of the adiponectin gene via direct interaction with the PPAR-γ/retinoid X receptor (RXR) via peroxisome-proliferator responsive elements (PPREs) present in the adiponectin promoter region [39]. Adiponectin-deficient mice do not show any improvements in atherosclerotic areas after PPAR-γ activation [40].

Incretin-based therapies, GLP-1 agonists, and DPP4 inhibitors offer optimal glucose-lowering effect without increasing the risk for hypoglycemia and weight gain [41]. Exendin-4, which is a GLP-1R agonist, upregulates adiponectin mRNA and increases secretion of adiponectin via a protein kinase A pathway [42]. Inhibition of DPP4 leads to an increase in the GLP-1 expression, which in turn enhances insulin activity. Sitagliptin and vildagliptin resulted in an increase in adiponectin levels in the current study, although its underlying mechanism remained unclear. Due to a significant effect on heterogeneity, Derosa’s study was excluded according to the sensitivity analysis. One possible reason for the effect might be the difference of population race between Derosa’s study (among Italian) and another four studies (among Japanese). Patient baseline level of HbA_1c_ in Derosa’s research exceeded 8.0% while other four studies were between 6.7 to 7.8%, indicating that baseline levels of glycosylated hemoglobin might present an effect on adiponectin in response to DPP4i treatment. Treatment duration might be the third cause of heterogeneity effect between Derosa’s study (12 months) and another four studies (3–6 months).

Sitagliptin or vildagliptin promotes secretion of adiponectin by enhancing the production of active GLP-1 [43]. Weight reduction or reduced BMI, waist circumference, and visceral fat accumulation may also elevate serum adiponectin levels [8, 44]. Sitagliptin induces a significant increase in adiponectin levels prior to weight loss, thereby suggesting that a reduction in visceral fat mass from long-term treatment causes changes in adiponectin levels [22]. Vildagliptin treatment elevates plasma adiponectin levels and this may have been due to a reduction in fat mass [45]. Speculations might be obtained that sitagliptin has no better specific selectivity on targeting proteins in the adiponectin-secretion pathway than active comparators. Oxidative stress can also cause dysregulation of secretion of fat-derived hormones such as adiponectin [46]. Sitagliptin or vildagliptin increases the expression of antioxidative enzymes, as well as promotes adiponectin secretion directly or indirectly by targeting signals of oxidative stress. The role of gliptins in oxidative stress must be investigated to better understand the mechanism underlying adiponectin secretion.

### Limitations

Our study has several limitations such as the inclusion of a small sample size as well as English-only clinical trials. Furthermore, unpublished results were not included, which in turn might cause potential publication bias. Also, changes in appetite or food intake were not recorded and the clinical trials were conducted for a short duration. Some heterogeneity was also observed in some of the results, although measures were taken to overcome them by performing a sensitivity analysis. Parallel evaluation of related adipokines was also not performed.

## Conclusions

DPP4 inhibitors promote the secretion of serum adiponectin in T2DM patients, thereby indicating that these have a cardioprotective effect. Further trials with larger sample size are needed to confirm the results and investigate the association between serum adiponectin levels and treatment of other DPP4 inhibitors. Long-term efficacy and cardiovascular endpoints should be studied to better guide patient treatment with or without other glucose-lowering agents.
